# A tamper-proof audit and control system for the doctor in the loop

**DOI:** 10.1007/s40708-016-0046-2

**Published:** 2016-03-19

**Authors:** Peter Kieseberg, Bernd Malle, Peter Frühwirt, Edgar Weippl, Andreas Holzinger

**Affiliations:** 1 SBA Research, Vienna, Austria; 2 Graz University of Technology, Graz, Austria; 3 TU Wien, Wien, Austria

**Keywords:** P4 medicine, Digital forensics, Manipulation detection, Data-driven science

## Abstract

The “doctor in the loop” is a new paradigm in information-driven medicine, picturing the doctor as authority inside a loop supplying an expert system with information on actual patients, treatment results, and possible additional (side-)effects, including general information in order to enhance data-driven medical science, as well as giving back treatment advice to the doctor himself. While this approach can be very beneficial for new medical approaches like P4 medicine (personal, predictive, preventive, and participatory), it also relies heavily on the authenticity of the data and thus increases the need for secure and reliable databases. In this paper, we propose a solution in order to protect the doctor in the loop against responsibility derived from manipulated data, thus enabling this new paradigm to gain acceptance in the medical community. This work is an extension of the conference paper  Kieseberg et al. (Brain Informatics and Health, 2015), which includes extensions to the original concept.

## Introduction and Motivation

While the concept of the “doctor in the loop” seems to be a logical consequence of the application of machine learning technologies and derived knowledge into medical science, one major problem arises: The doctor in question is forced to trust the results derived from algorithms based on the authenticity of stored data to a large extent, while being seen as the primary responsible party during information provisioning, as well as during treatment, i.e., the doctor retains responsibility or, in case he/she is involved in the selection of the source data, even gains more, while loosing control over the process. With the technology available to tackle large amounts of complicated data in real time through Big-Data techniques, results derived from such processes may even become more uncontrollable. This opens up the problem of acceptance of the “doctor in the loop” approach by medical personal. The question is the trustworthiness of the underlying data and execution chains, especially considering manipulation, e.g., in the aftermath of a wrong treatment:

- In case of errors on the doctor side, he/she could try to cover the tracks by changing the wrongful data that led to the treatment.
- On the other hand, as the doctor is seen as the responsible person that is going to be blamed in case of errors, he/she needs to be ensured that in case the wrong suggestions came from the system, he/she is protected against legal actions. Safeguarding the doctors is especially important, since the whole concept relies on their participation [2].
- Securing the system against manipulation is especially important in order to generate trust in the system on the side of the patients and the health care providers. Furthermore, as a doctor in the loop systems would logically constitute an ICT-critical infrastructure, data manipulation could be a possible threat in scenarios of cyber-crime (e.g., illegal drug acquisition) or cyber-terrorism (e.g., by seeding distrust toward entities in the national health system).

Thus, in order to mitigate these risks for the overall concept, manipulations in the underlying database need to be detected, as well as control over the information entered by the doctor needs to be safeguarded against subsequent manipulation. This also includes the manipulation-secure logging of execution chains of enrichment and analytics algorithms and workflows. The contribution of this work can be summarized as follows (see also [1]):

- We provide a model of the “doctor in the loop concept” including an abstract architecture of its entities with respect to security.
- Attack scenarios and attacker models against this approach are devised.
- Based on these models, strategies for mitigation are defined.

Compared to the conference paper, the following extensions are provided in this work:

1. Extension of the approach to multiple decision makers (see Sect. 3.3).
2. Adaption of the approach in order to be suitable for closed-source Database Management Systems (DBMSs) and environments (see Sect. 3.4).
3. In-depth discussion of limitations and possible countermeasures (see Sect. 4.3).

## Background and related Work

### Experts in the loop and medical databases

Interactive machine learning has been a very popular topic in research throughout the recent years, especially considering the medical domain with its vast amount of applications in the sectors of diagnosis, as well as treatment. In [3], the authors provide a comprehensive comparison on different training algorithms for supervised machine learning, mainly focusing on the aspects of speed, accuracy, and scalability. Focusing on the pure algorithmic level, matters of security and especially data protection are left out. Alongside this work,  [4] discusses how computer-assisted presentation of case data can help experts to infer machine-implementable rules for case definition in electronic health records. Here, the authors apply an expert in the loop approach and demonstrate the usefulness of their techniques with a practical medical example on acute liver dysfunction (ALD). Furthermore, a multitude of applied work that utilizes data mining and (interactive) machine learning in medical research has been proposed in the recent past like [5] for the prediction of heart diseases, or [6] discussing possible applications in radiology. In [7], the authors consider not only the benefits, but also the challenges when mining electronic health records (EHRs).

Regarding sharing of information in medical databases, in [8] the authors discuss the effects of the American Recovery and Reinvestment Act of 2009 on medical research, namely the production of large-scale databases of patient data allowing better and larger studies. While acknowledging the need for removing protected patient information, the paper focusses more on the benefits of data sharing without acknowledging malicious intent. Various works exist on the problem of de-anonymization of health records (e.g., [9]), which is thus not in the focus of this work, since the architecture proposed in this work allows the application of various kinds of additional countermeasures in order to protect patient privacy. In their work [10], the authors propose a practical framework under development that makes recent developments in the realm of machine learning accessible to practitioners. This is based on their observation that despite the fundamental developments on the theoretical and conceptual side, adoption of machine learning techniques by practitioners has been low. Still, while providing many practical considerations, the topic of security and protection of sensitive data was only touched slightly in this concept.

The problem of securing infrastructures relying on human behavior has been discussed throughout the last decade and more, being one of the very fundamental problems for computer security [11]. The problem is often related to the issues of awareness [12] or missing usability in security [13], as well as other subtopics, also including the sharing of data between different entities [14] where each participant has his/her own agenda in dealing with the supplied data. It has to be kept in mind that research data forms a very valuable resource for many research laboratories. This is also often related to the issues of providing health-related information to other clinicians [15] or to automated systems [16], where the original owner of the data loses control over the further dissemination. In [17], the authors identify security and privacy issues as one of the major open research issues in the development of medical cyber-physical systems (MCPS), especially considering that interoperability capabilities will open up new attack surfaces that can be misused to harm patients by, e.g., manipulation of data or direct access to critical system components.

### Chained witnesses

The term “chained witnesses” was coined in [18], where the authors propose a technique for securing internal mechanisms of databases against manipulation. The main advantage of this approach over the multitude of approaches described in the literature was resilience against an attacker model that included the database administrator as possible adversary. While this is discussable in most real-life systems where the database administrator is seen as a trusted entity, this is especially interesting in the “doctor in the loop” concept.

The main principle of this approach lies in appending a so-called *witness* for each transaction that is issued against the database to the internal logging mechanisms: The database storing the information is considered as untrusted; furthermore, even file system administrator rights are assumed for the attacker. Let $D_i$ be the* i*th data record written to the database at time $t_i$. Furthermore, we assume that $\mathcal {H}$ is a cryptographically secure one-way hash function, $\mathcal {T}$ is a trusted third party, and $\mathcal {R}$ is a secure pseudo random number generator (PRNG) and $r_i$ is the result of its* i*th iteration. The witness for transaction $D_i$ is calculated as

$$\begin{aligned} w_i=\mathcal {H}(w_{i-1}||D_i||t_i||r_i)=\mathcal {H}(w_{i-1}||D_i||t_i||\mathcal {R}(r_{i-1})) \end{aligned}$$

with || denoting string concatenation. The tuple $(t_i, w_i)$ is then called the *signature* of the record $D_i$. In order to start the hash-chain, an initialization phase is required: A trusted third party $\mathcal {T}$ selects a random number *s* as seed for the PRNG and thus generates $r_0$ by using the PRNG on *s*. Furthermore, the initial witness is defined as $w_0:=\mathcal {H}(r_0)$.

**Fig. 1:**
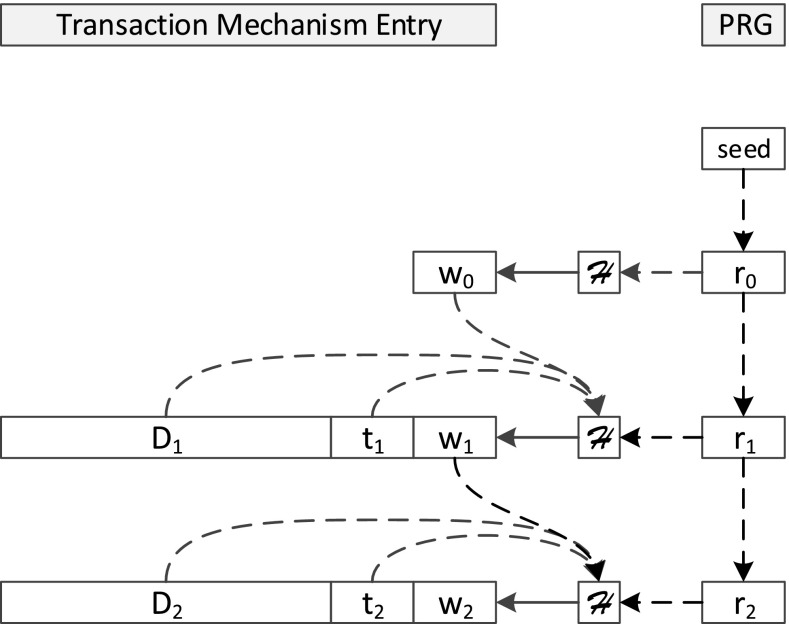
Construction and storage of the chained witnesses

Due to the definition of the witnesses as chained hashes, any changes in older datasets lead to cascading changes in all subsequent witnesses (Fig. 1 shows the chaining). For the verification, the data of the protected internal logging mechanisms are executed against an old trusted backup under the premise of $\mathcal {T}$ and compared to the investigated database instance. This also works, when reverting the whole database to an old state: The PRNG is seeded with a seed unknown to the attacker, every iteration changes the state of the PRNG, which cannot be calculated backwards (this is ensured by the criteria for the hash function and the PRNG in [18]). Thus, in the case of reverting, the states of the missing entries can be detected easily. In [18], the authors propose several mechanisms for achieving this kind of manipulation security in real-life environments, especially targeting internal database mechanisms for providing rollbacks (so-called  *transaction logs* 2). The transaction log is stored directly by the database environment as an internal component. Figure 1 also demonstrates, how the log entry is extended in order to store the witness, more details on the exact specification of the log entries can be found in [18]. Furthermore, the database management system (DBMS) must be modified in a way to provide the calculation of the respective witness as an atomic action, invisible to and uninterruptable by the administrator, i.e., the mechanism for writing the transaction log needs to be modified directly in the DBMS-code in order to fetch the random numbers $r_i$ and calculate the witness immediately, without leaking $r_i$ to the administrator. As shown in [18], the implementation of such a process can be done for MySQL; furthermore, the authors pointed out solutions for closed-source DBMSs based on the database replication logs.

### The doctor in the loop

**Fig. 2:**
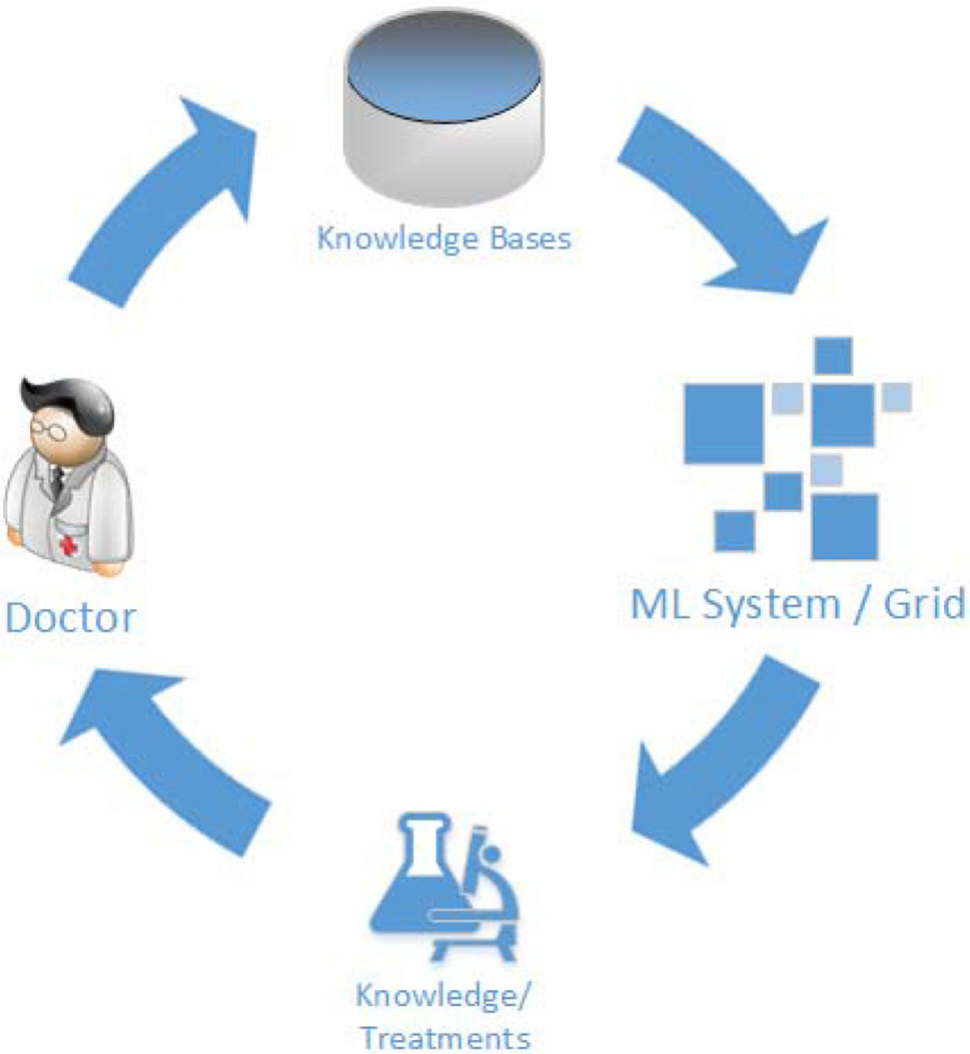
The doctor in the loop

The concept of the “doctor in the loop” is an extension of the increasingly frequent use of knowledge discovery for the enhancement of medical treatments together with the “human in the loop” concept: The expert knowledge of the doctor is incorporated into “intelligent” systems (e.g., using interactive machine learning) and enriched with additional information and expertise. Using machine learning algorithms, medical knowledge and optimal treatments are identified. This knowledge is then fed back to the doctor to assist him/her (see Fig. 2).

While general techniques regarding data-driven research have their own problems with respect to privacy protection (see e.g., [19]), an additional major problem for the doctor in the loop lies in guaranteeing the trustworthiness of the data provided by other entities and by analysis workflows. Furthermore, the data provided by the doctor need to be secured against subsequent manipulation in the case of a cover up, either by the system, or by the doctor him-/herself. In this work, we will solely focus on this problem and leave the problems of privacy protection and data leakage discovery to the literature [14, 20].

## The Approach

The approach outlined in this section is based on the generic concept of the “doctor in the loop” as described in Sect. 2.3. In order to motivate the chaining approach, we will define the entities and their relations, including the chaining mechanism.

### Entities and relations

**Fig. 3:**
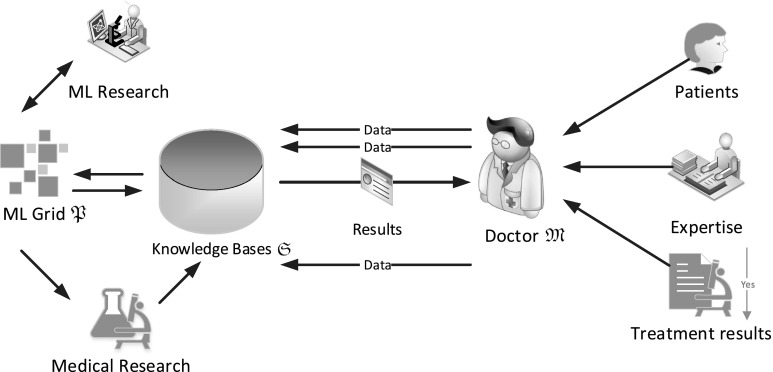
Entities and Relations

For our analysis, we define a more specific model for the doctor in the loop. Figure 3 gives an overview on the components:

- The *Doctor*, who is the main expert in the cycle, collects data from patients, including their reactions to individual treatments and potential other effects. Furthermore, he/she provides additional knowledge from his/her experience and sanity-checks results. All data he/she provides to the system are sent to the Knowledge Base, which also provides him/her with the relevant feedback. In the basic approach, we resort to a single doctor entity, which is rather uncommon in real-life environments, the required extensions to the chaining can be found in Sect. 3.3.
- The *Knowledge base* provides the store for the data and all results of workflows and external resources, as well as the only means for communication between the doctor and the other entities. This entity is the primary target for our chained witnesses approach, since all data that are transferred between the relevant entities for the “doctor in the loop” approach utilize it. The knowledge base may also host stored procedures for the analysis of the data, i.e., parts of the ML-grid are implemented as stored procedures inside the knowledge base.
- The *Grid* serves as a generic model for a machine learning/reasoning structure that takes input data and returns results using analytics algorithms. The grid may be implemented as external mediation tool, as well as in the form of internal stored procedures inside the knowledge base. In our concept, the exact definition of the grid will be kept on an abstract level, since providing manipulation security will be done on the side of the underlying database of the knowledge base.
- *Interfaces* from other entities to the knowledge base are logged by the underlying DBMS. This includes all transactions changing data or structures in the database, as well as the change and invocation of stored procedures that may implement part of the grid.
- The entity *Medical research* denotes external knowledge bases that serve as external data input to the grid, or to the knowledge base.
- *ML research* provides the grid with new algorithms for the analysis of the data stored in the knowledge base.

### Interaction and chaining

For the abstract approach, we only consider a general scenario where a generic data receiving decision maker $\mathfrak {M}$ (e.g., the doctor) sends data to a generic data store $\mathfrak {S}$ (e.g., the knowledge base). Furthermore, an entity $\mathfrak {P}$, the data provider, operates on the same database and delivers a result to $\mathfrak {S}$. $\mathfrak {M}$ takes a result (e.g., a treatment) based on the data in the database and returns additional information, especially on the reaction of the patient and other (side-)effects. Furthermore, $\mathfrak {M}$ controls the results stored in $\mathfrak {S}$ with respect to sanity-checks based on his/her background knowledge and issues respective corrections to $\mathfrak {S}$ that are subsequently used by $\mathfrak {P}$. From a security point of view, this especially implies that the exact order of the transactions with respect to the knowledge base is of vital importance in order to guarantee authenticity.

#### Data provider

The model of $\mathfrak {P}$ is selected to be as generic as possible and covers all single data providing entities except the decision maker. This especially includes all parts of the grid, as well as additional data sources with respect to Sect. 3.1. Due to the assumption that $\mathfrak {P}$ might be some proprietary entity, incorporating additional mechanisms for controlling the decision provider(s) is not reasonable. Furthermore, $\mathfrak {P}$ might in reality consist of several different entities (internal stored procedures and external workflow engines), i.e., $\mathfrak {M}$ might provide data to and receive information from several different data providers $\mathfrak {P}_i, i\in \mathbb {N}$. Thus, the $\mathfrak {P}$ only needs to fulfill the following pre-requisites:

1. All results are written to $\mathfrak {S}$, there is no additional side channel to $\mathfrak {M}$, i.e., $\mathfrak {M}$ and $\mathfrak {P}$ are independent.
2. Everything sent to $\mathfrak {S}$ by $\mathfrak {P}$ is signed using state-of-the art cryptographic technologies and is therefore assumed to be unforgeable.

Especially requirement two seems to be strong, still this is a standard requirement in many current communication protocols.

#### Data store

The data store possesses an internal table structure for storing all collected data, invoked enrichment algorithms, as well as the received data, protected with the chained witnesses approach: For each entry in the transaction log $D_i$, the respective signature $(t_i, w_i)$ is stored (see [18]). It must be kept in mind that the only connection between two entries $D_i$ and $D_j$ lies in their timely succession, i.e., all changes in all tables are stored in the same transaction mechanism, ordered by the time of entering $t_i$. This also holds true in case of several decision makers $\mathfrak {M}_i$. In the **setup phase**, the initialization is done by a trusted third party ${\mathfrak {T}}$ (see below). We furthermore assume that the data store is run independently from the underlying physical server, i.e., $\mathfrak {S}$ possess administrator privileges over all tables, as well as full access to the file system for enrichment and processing of incoming and outgoing data, as well as for restructuring the database layout (tables, views ...), including full control over log settings. Still, it does not possess root privileges on the underlying machine, which is run by ${\mathfrak {T}}$ or another trusted entity. In addition, the data store frequently sends a backup to ${\mathfrak {T}}$, which is validated as shown below. The newly validated database image iteratively serves as the new base point for the next validation cycle.

#### Decision maker

The decision maker $\mathfrak {M}$ is independent from the data store, i.e., it does not have any control over $\mathfrak {S}$. Furthermore, it is also independent from all data providers (see there). In this approach, we assume that the decision maker is honest in general (see data insertion).

#### Trusted third party

The trusted third party ${\mathfrak {T}}$ controls and manages the random values needed in the chained witnesses approach for the data store. During the setup phase, a new random seed *s* is selected and the first random value $r_0$ is generated using the cryptographically secure pseudo random number generator (PRNG). Furthermore, the first witness $w_0=\mathfrak {H}(r_0)$ is sent to $\mathfrak {M}$. Additionally, ${\mathfrak {T}}$ can be the entity responsible for running the physical server for $\mathfrak {S}$, including root privileges. While ${\mathfrak {T}}$ is thus in a very powerful position, ${\mathfrak {T}}$ must be independent from all other entities, especially from all data providing parties, thus possessing no interest in data manipulation. Furthermore, interaction between $\mathfrak {S}$ and ${\mathfrak {T}}$ is limited to the setup phase and during the verification of authenticity.

#### Network providers

The network provider is responsible for enabling the communication between the data provider and the decision maker. We assume that all traffic is protected by end-to-end encryption against eavesdropping, other attacks by a malicious network provider, e.g., denial of service, are not inside the scope of this paper. This also holds true for the underlying public key infrastructure that is needed in order to facilitate the encrypted communication.

#### Data insertion

The decision maker is modeled to receive data from outside machine-based systems, especially by the patients during personal consultation. As outlined later in the attacker model in Sect. 4.1, we assume that the decision maker is in principle honest, i.e., at the time of consultation, no harm toward the patient is intended from his/her side. This also means that the data entered into the database are correct at the time of insertion. All data received by the patients are immediately stored to $\mathfrak {S}$.

#### Verification of authenticity

In the verification step, ${\mathfrak {T}}$ extracts the internal transaction logs (this is possible using a method provided in [21]) and uses a trusted backup as starting point for consecutive execution of the log entries, thus verifying the witness for each transaction by using the secret initialization vector *s* and the PRNG. The first encountered invalid witness provides the position of a manipulation of the log. Furthermore, the result of the verification is compared bit-wise to the current database, thus being able to uncover changes done directly in the underlying file system.

### Multiple decision makers

**Fig. 4:**
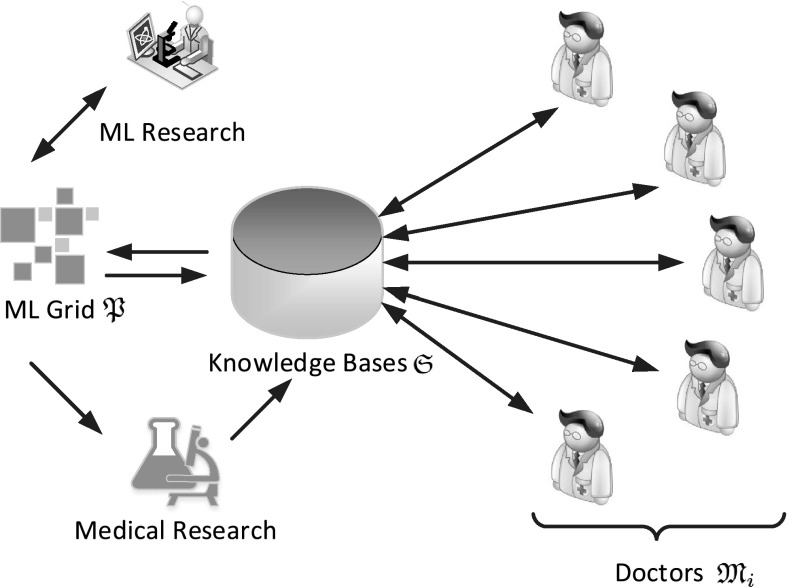
Multiple doctors in the loop

The basic approach is very limited with respect to the human interfaces, i.e., it only considers a single decision maker $\mathfrak {M}$ (e.g., one doctor). This approach, while reasonable for demonstrating the fundamental chaining mechanism and providing a fundamental security analysis, has a very large drawback. In a real-life environment, e.g., a research lab for biomedical research, several decision makers $\mathfrak {M}_i$ will be involved for different reasons: The advice of experts on different medical fields might be needed, as well as different experts from the same field in order to reduce errors and enhance accuracy (see Fig. 4).

We assume without loss of generality that all $\mathfrak {M}_i$ use the same interfaces to the database, being capable of changing the same tables, attributes, and even records. This is done in order to formulate the problem of attribution of changes as general as possible and allowing the greatest amount of inferences between the decision makers, also including malicious collaboration. While secrecy of the data entered by the $\mathfrak {M}_i$ is not an issue in this architecture, vital importance is put on the aspects that no decision maker (or any combination of $\mathfrak {M}_i$) is capable of (i) hiding his/her/their changes, (ii) planting changes as if they were issued by someone else, or (iii) changing the logged order of changes with respect to the application order (i.e., a transaction $T_i$ is logged after $T_j$ with $i<j$).

In order to implement this chaining into the architecture, the following pre-requisites are assumed:

1. All decision makers $\mathfrak {M}_i$ use different user profiles for accessing the database.
2. The attribution of the statement to the respective user issuing it can be done on the DBMS-internal level, i.e., the transaction mechanism has access to the actual user issuing the request. This is typically available for DBMSs like MySQL with InnoDB in order to provide access control. This also means that the database administrator cannot control the attribution of statements to the log.
3. The same holds true for the database replication mechanism in case of a closed-source DBMS, see Sect. 3.4.

**Fig. 5:**
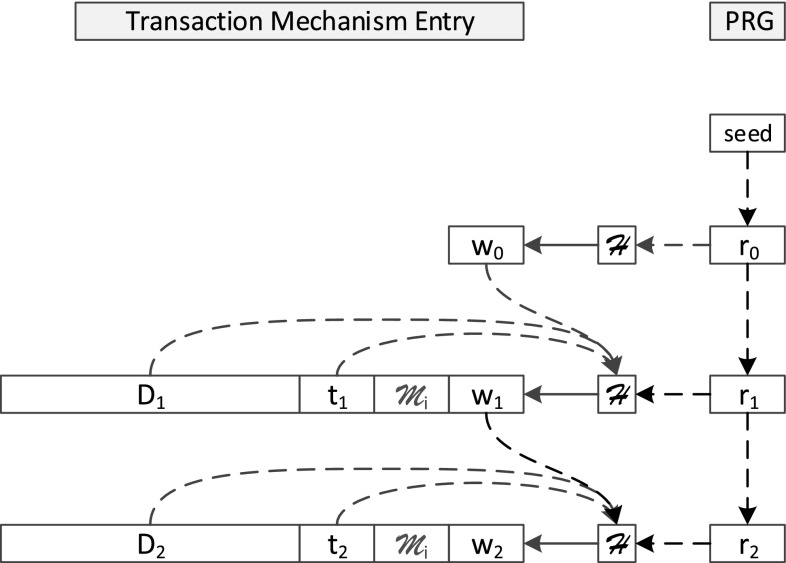
Modified chained witnesses for multiple $\mathfrak {M}_i$

The calculation of the chained witnesses can then be extended to account for the attribution mechanism to specific users by including the user-id $\mathfrak {M}_i$ for the *i*-th user into the chaining (see also Fig. 5). The witness for transaction $D_i$ is calculated as

$$\begin{aligned} w_i= & {} \mathcal {H}(w_{i-1}||D_i||t_i||\mathfrak {M}_i||r_i)\\= & {} \mathcal {H}(w_{i-1}||D_i||t_i||\mathfrak {M}_i||\mathcal {R}(r_{i-1})) \end{aligned}$$

In MySQL, the user-id $\mathfrak {M}_i$ can easily be included by a very simple rewrite in the logging routines: Since the InnoDB storage engine provides ACID-compliance, the user is known to the transaction mechanism, the linking is thus quite simple to implement.

### Adaption to closed-source environments

**Fig. 6:**
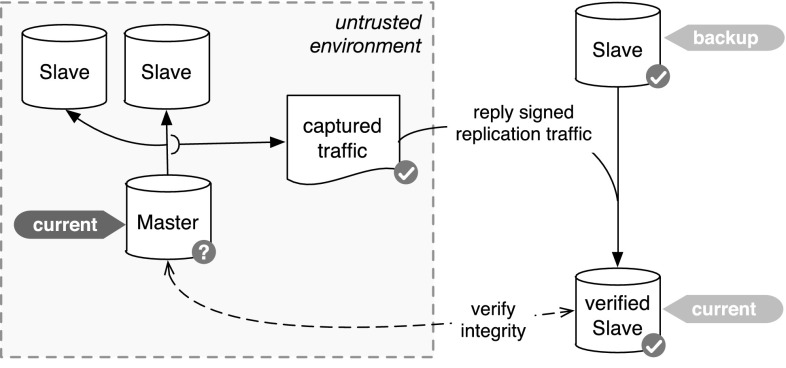
Verification with replication ([18])

Unfortunately for the original approach, a lot of systems typically accessed by doctors and put to work in medical research environments are not fully open source. Especially regarding high-performance database management systems, the major vendors like Teradata, IBM (DB2), and ORACLE stay closed-source. Thus, contrary to the scenario in case of using open source alternatives, changes directly to the transaction mechanism are neither feasible nor practical. In order to employ the original concept of the chained witnesses for the doctor in the loop, internal mechanisms that have a passive, read-only interface to the outside world need to be employed. As outlined in [18], the database replication mechanism is a suitable choice for supporting the approach: The task of this mechanism lies in mirroring a database instance, typically referred to as *master instance* to several so-called *slaves*, instances that are exact copies of the original database. Typically, this mechanism is employed in order to generate redundant copies for so-called *hot backups*, allowing the possibility to switch from the master instance to a slave instance transparently, e.g., in the case of defects on the master instance. Thus, the information that needs to be stored in the slave instances is typically not limited to the datasets alone, but includes vital information on the structure, large objects and even metadata like session information and timestamps. It is thus possible to adapt the chained witnesses approach to this interface, as shown in Fig. 6.

In this approach, the data stream from the master instance to the slave instances is captured and the witnesses are added right before sending the stream to the slaves. This requires that the whole signing procedure is added in an extra node right on the transport layer. Since the data stream of the data replication mechanism is typically not documented for many closed-source DBMSs and might change between releases, the information is not decoded but the whole stream is simply split into blocks of a pre-defined length. These blocks are then chained using the original approach, still, due to the need to control an active slave instance for verification purposes, the trusted third party ${\mathfrak {T}}$ is far more involved compared to the original approach using the transaction mechanism:

- Since the replication mechanism extracts all changes to an actual separate database instance instead of some internal mechanism stored on the same machine, attacks by the root of the master instance need to take place when the data are written, i.e., even for an attacker with root privileges, it is not possible to later on alter the data that were submitted to the slave instance under the control of ${\mathfrak {T}}$.
- Internal transaction mechanisms are typically limited in terms of disk space that is provided for them and older entries are removed after a while. While this is no problem per se in the original approach, since the chaining still needs to be valid, and regular audit of old entries allows (i) the removal of such old entries without implications to the verifiability and (ii) reduce the workload needed to be carried out in the case of a check for manipulations, using the replication mechanism allows for a much more transparent implementation, where no changes are deleted and the whole history of the original database can be rolled back.
- Since the replication mechanism is made for reliable mirroring of databases, it can be accessed very easily and without tinkering with the DBMS in place. This is not only interesting in the case of closed-source approaches, but also reduces the implementation overhead in case of switching to a new version of the same DBMS. While the transaction mechanism as an internal function is typically undocumented and can be altered between even minor version changes, the interfaces to the replication mechanism will stay much more stable and changes will be documented well.

The main drawback of this modification compared to the original approach lies in the effort on the side of the trusted third party ${\mathfrak {T}}$: Instead of storing a random seed and only having to become active in case of manipulation detection or maintenance, ${\mathfrak {T}}$ has to operate a full instance of the DBMS with at least the same performance capabilities with respect to data insertion. Furthermore, in case of commercial platforms, this also includes additional costs for licenses, as well as trained personnel for operation. This also hinders outsourcing this task to a central authority that provides the services of a trusted third party to several different operators.

## Evaluation

### Attacker models and attack vectors

In this section, we give a description of the attacker models and attack vectors with respect to the assets of the “doctor in the loop” approach.

#### Data provider and decision maker

Both entities could have the interest of manipulating data on the data store in case of cover ups, e.g., manipulating previously delivered incorrect data. The main attack vector of the decision maker lies in updating data on the data store, provided either by itself, or as result (treatment) data from the data provider. The data provider possesses the same attack vectors, but in addition, he/she might try to manipulate and/or re-execute stored procedures that operate on the data in order to cover up wrong results. In order to keep the concept as simple and strong as possible, we assume that there is no dedicated secure application controlling access to and from the database by the entities, i.e., the entities write their changes directly into the data store. This is especially important in order to be secure against SQL-injections or related attacks by default.

#### Data store

The data store itself is an important entity in the overall concept, since it serves as the central data exchange platform and is thus vital for providing trust in the “doctor in the loop” concept. The database administrator controls all access to the database, including the possibility to undo logs, as well as change arbitrary data and structures. Furthermore, not only the database itself, but also the underlying file system, can be of interest for an attacker: As outlined in related work [21], file carving techniques can be used in order to retrieve or manipulate data by directly accessing the database files on the file system. In this evaluation, we thus concentrate on these two fundamental attack vectors:

- The *Database administrator (DBA)* possesses administrator privileges on the database itself, including the ability to change logging routines and user rights, as well as read access to the underlying file system.
- The *File system administrator (FSA)* can modify arbitrary files on the server, including the files belonging to the database, as well as the OS (system) logs. He has no access to the database query interface though.

Neither of the two attackers possesses root privileges on the respective database server.

### Security evaluation

In this section, we will analyze the respective assets that could be targeted by the attackers modeled in the previous section.

#### Manipulation through the database (All except FSA)

Both, the data provider, as well as the decision maker could be interested in reissuing incorrectly entered data. In case they act with their own privileges, i.e., as data provider or decision maker, every modification of data is stored in the internal logs, together with the respective timestamp of the change, making it easily detectable. In case the attacker possesses administrator privileges on the DBMS (DBA), the internal log mechanisms are under the full control of the attacker, except for the chaining: Since the attacker still does not possess root privileges on the server, it is impossible for him/her to read the value $r_i$ from the RAM, which is then used in the generation of the witness with $\mathfrak {H}$. Since $\mathfrak {H}$ is a cryptographic hash function, when given $h:=\mathfrak {H(h')}$, $h'$ cannot be deduced from *h*. This also holds true for the closed-source approach, the hashing is still done the same way, the main difference lies in the fact that the chained blocks are not aligned with logical borders of statements, but using a fixed block size.

#### Targeting stored procedures (DBA)

The database administrator can execute and modify every stored procedure on any stored dataset. Still, in case executions change any data in any table in the database, the changes are logged in order to retain transaction safety. Since data replication is used to provide hot backups, these changes are immediately sent to the slave nodes.

#### Manipulation through database files (FSA)

The file system administrator could bypass all logging mechanism by manipulating data directly in the underlying database files. This includes the transaction log and other rollback mechanisms, which have to be invoked by the DBMS. Using the witnesses, these changes remain detectable, since the resulting database in the verification step will be different from the one currently on the server. Still, the attacker could insert data via the file system and remove it right before the validation, making this manipulation undetectable. As a countermeasure, the validation process should be done frequently at random times. Furthermore, we propose to use the chaining witnesses approach with respect to special logs containing checksums of the database files. This attack can be omitted in case of the closed-source approach, since the attacker does not have access to the database files of the slave instance under the ownership of ${\mathfrak {T}}$.

#### Manipulation of the DBMS

The attacker could remove the chaining witnesses from the source code of the DBMS and install a recompiled version. While this is possible, this action would require root privileges on the server. Furthermore, modifications on the binary could be easily detected via frequent comparison of checksums of the respective code to the originally issued version.

#### Modification of the transaction mechanism (DBA)

The authenticity of the information in the transaction mechanism/log is protected by the chained witnesses approach, so every manipulation can be detected under the given attacker model and the manipulated record can be identified. This could only be circumvented by deleting the whole log, which itself is an highly obvious manipulation pointing to the database administrator.

#### Combined attackers

In the above examples, we split the attacker between the DBA and the FSA, still, the resilience of the approach is retained even in case the attacker possesses both privileges. This can be directly inferred from this section, since the chaining is done on DBMS level, without the involvement of either, the DBA or the FSA.

### Limitations

The limitations of the proposed approach can mainly be attributed to limitations of the original chained witnesses approach, especially regarding the lifetime of the internal transaction logs and problems related to an attacker possessing root privileges: Since such an attacker is expected to have full control over every aspect of the system, the following additional attack vectors are available that the approach is vulnerable to

- *Modification of the DBMS* In case of root privileges, the attacker can simply modify the DBMS in such a way as to either remove the chained logging altogether (which is rather obvious and easy to spot), or by modify the chaining in order to give exclusive powers of manipulation: For example, the attacker could replace the hash function $\mathfrak {H}$ with a weaker version, or take control over the generation of the random numbers $r_i$, both allowing him/her to later on manipulate entries, while still using the protection mechanism against other entities.
- *Control over memory* With full control over the memory of the machine the DBMS is running on, the attacker could simply record the sequence of random numbers $r_i$ and thus manipulate older entries. While this is possible in theory, the attack possesses one issue in real implementations: The changes must be done on the transaction log by removing all current entries down to the point of manipulation and then recreating them using the sequence $r_i$. This can be a problem in case of running databases in case of rollbacks or crash recoveries during the manipulation process.
- *Control over interfaces* Especially considering the closed-source approach with full control over all interfaces, the attacker is capable of manipulating the data streams sent to the slave instances, while making no changes on the master instance.

Furthermore, the approach only works with DBMSs that actually provide transaction safety or data replication and thus provide the respective mechanisms.

More specific to the architecture provided in this paper, the main limitation lies in the assumption of independence of the different entities, which in reality may not be guaranteed due to the setup of the overall environment (e.g., a hospital running a “doctor in the loop” approach might control the doctor, the database and parts of the grid, as well as ${\mathfrak {T}}$). Against attacks arising from this overlap in duties and authorities, managerial countermeasures regarding organizational security must be employed, starting with strict separation of duties and reliance on an external party ${\mathfrak {T}}$.

## Conclusion and Future Outlook

In this work, we provided an approach for protecting decision-relevant data in a generic “doctor in the loop” setup against manipulations targeting the underlying database, including closed-source database management systems. This is especially needed in order to increase trust in the “doctor in the loop” concept for both sides, the involved medical personnel, as well as external partners and research labs providing results based on the data. In order to allow for a real-life audit and control system, we additionally provided means for attribution of changes with respect to several decision makers (doctors).

Future work is especially needed in the area of usability in order to effectively incorporate the architecture into the daily routines without introducing even more overhead for the medical personal, thus enabling the “doctor in the loop” to use the benefits of machine-supported medicine. Future work from our side includes the development of a prototype implementation in order to test the effects of introducing this concept into real-life environments, specifically targeting the scientific field of biomarker research.

## Footnotes

2 It must be noted that the term “logs” is slightly misleading, since these are not human readable log files, but internal mechanisms for ensuring transaction safety
